# Aneurism
*Mid. Medical and Surg. Report, No. vii.


**Published:** 1830-07-01

**Authors:** 


### XLIV.

### WORCESTER INFIRMARY.

### Aneurism.*

Case 1. *Aneurism, Ligature of the Femoral Artery—Amputation—Recovery.*

John Milner, æt. 30, admitted Oct. 3, 1829, under the care of Mr. Pierpoint, with a pulsating tumour extending from above the middle of the thigh to the knee, and the skin suffused with an inflammatory blush; an opening in the inner part of the tumour about the size of a shilling, disclosing a dark speck of coagulum pulsating perceptibly; œdema of the limb extending nearly to the groin; pulse very languid; some cough; sallow and anxious countenance.

Is a hard working man, and accustomed to lift great weights. Thinks himself healthy in general, but has had asthma from his birth. About five weeks ago, he was attacked with pain just above the knee, extending to about the middle of the thigh, where he said there was a small hard lump, in which he felt a beating, particularly when he pressed his hand upon it, in the act of milking. He says that ever since a kick which he received from a horse, 20 years ago, there has been a tumour about the size of a large pea, about the middle of his thigh. Had advice for it a month since. Leeches and blisters were applied at different times. The thigh was rolled up, under the pressure of which it began to swell exceedingly; six days since, supposing the tumour to be an abscess, a lancet was introduced, and a little coagulated blood came away, and on the following day the wound burst, and about three pints of blood gushed out, and then stopped spontaneously. The wound was then closed, and the limb bound up exceedingly tight.

At 3 p. m. next day, the femoral artery was tied about an inch below Poupart's ligament, and the wound was united by sutures and sticking plaister. *A poultice to the ulcerated opening, and an anodyne in the evening.* The ano-

* Mid. Medical and Surg. Report, No. vii.

dyne was repeated, he passed a good night, and was better on the 5th. On the 6th, grumous discharge issued from the opening in the tumour, and on the 7th it was very copious—no pulsation in the limb, which was much less swollen, since the operation. 8th, Great discharge from the inferior opening—superior one pale and without much union —œdema decreased—purged. *Mist. cret. c. tinct. op.* ♏ x. *conf. arom.* ℈j. *post sed. liq.* The purging ceased and the upper wound improved. On the 10th the œdema of the thigh nearly gone; a counter-opening made two inches above the ulcerated wound, and "an amazing quantity of pus discharged." 11th, Portions of coagulum evacuated from both lower openings—wound in the groin looking healthy. *Quinine draught every six hours—meat and beer —gentle bandaging.* On the 14th the lower openings laid into one. Next day the coagulum was found protruding through the lower wound, and its removal was followed first by a large quantity of pus; and then by a gush of arterial blood, which continued to well out of the wound. The hæmorrhage was restrained by stuffing the wound with lint. Amputation was now determined on in consultation, and immediately done, close to the trochanter major. Six vessels were tied, the internal circumflex was very large and bled profusely, and the patient was so exhausted, as to require laudanum, and brandy and water. The aneurismal cavity extended from the inner condyle to three inches up the *stump* between the muscles; there were hardly any remains of a sac; the internal coat of the popliteal artery was much diseased, and a ragged opening existed in its upper part; the bone for the space of three or four inches was denuded and subjected to considerable absorption; and finally, great destruction had taken place in the soft parts around it. Stump dressed with sticking-plaister and chalk cerate. On the 17th he was ordered infusion of roses and quinine, and next day, on the first dressing, partial union had taken place. 19th, Discharge from stump profuse; ale and egg for breakfast. On the 21st, the ligature came away from the upper wound; healthy granulations on the stump. On the 29th, the upper wound was perfectly healed. On the 2nd Nov. "general union" of the stump. A small sinus formed at the upper part of the stump, and a piece of bone died and was removed, but with the exception of these accidents, and a slight fall which he experienced, no untoward circumstances obstructed the gradual progress to recovery, a desirable consummation that took place on the 31st Dec. when the patient was discharged cured.

The foregoing case is a happy illustration of good surgery and bad. The latter was well exemplified in the treatment of the case before its reception in the hospital, the former in its subsequent management. There is one point however, of which we are disposed to question the propriety, and it is this; on the patient's admission, the diffused aneurismal swelling extended from the knee to above the middle of the thigh, and for this the "femoral artery was tied about an inch below Poupart's ligament." Under these circumstances it cannot be doubted that the ligature was placed on the common femoral, above the origin of the profunda. We need not proceed to explain that this operation would be much less calculated to obviate hæmorrhage from an opening in the popliteal artery, than tying the superficial femoral itself; and, under correction, we submit that sufficient space existing for this operation, it ought to have been adopted in preference to that which was actually performed. The improper puncture of the sac, reduced the case very nearly to one of wounded artery, and we all know the practice in such a case is, to tie the vessel above and below, or if that be unattainable, to secure the upper portion as closely to the wound as circumstances will permit.

CASE 2. *Aneurism of the ascending portion of the Arch of the Aorta.*

Joseph Freeman, 57 years of age, a sawyer by trade, in the habit of working in the pit; a particularly muscular, stout, thick-set man, of the ordinary stature, and who had been a hard drinker, was admitted into the Worcester infirmary, September 13th, 1828. Complains of pain in the right side of the chest, much augmented on inspiration, with urgent dyspnœa and cough. Pulse full; tongue foul; bowels pretty regular. There is considerable dilatation, and increased pulsation of the arteries about the upper portion of the same side; and a pulsating tumour, near the size of a pigeon's egg, at the intercostal space between the second and third rib, is distinctly felt, and perceptible, also, to the eye.

From his occupation, he has been in the constant practice of moving very heavy pieces of timber; but has no recollection of the receipt of any particular injury, from which he could date his present affection. He states that he was first seized about twelve months or more ago, previous to this time, with shooting pains in the right side of the chest, and weakness of the right arm; these symptoms he attributed to cold, and still continued his employment, for perhaps four or five months, being bled for the pain twice at his own request. Finding his disease getting worse, he at length consulted a surgeon, who prescribed frequent topical bleedings, by the aid of leeches, a very abstemious diet, especially avoiding all stimulating liquors, with perfect quietude; from such treatment he derived much benefit. Soon after this, he obtained a letter for the infirmary.

On his admission, he was cupped on the right side, and took a saline aperient mixture every four hours. Low diet. The cupping very materially relieved his breathing; but in a few days, the pain in the side much increased, with accelerated circulation; for the relief of which, leeches were applied, and a mixture, containing the tincture of digitalis, administered three times in the day, keeping the bowels regular, with five grains of the cathartic extract at bed-time. The vinum colchici was also given in conjunction with the tincture of digitalis, and leeches very frequently had recourse to: blisters, too, were used on several occasions.

On the 3d Nov. he had much pain in the tumour and dyspnœa, with troublesome cough and rattling in the throat (a very prominent symptom since his admission.) Eight leeches were applied, and afterwards a seton, but the latter aggravated the distress and was withdrawn. Narcotics were given and particular symptoms met as they arose, whilst spare diet and absolute rest were strictly enjoined. The disease made gradual but steady progress during the Winter, and in February the tartar emetic ointment was rubbed on his side, whilst tincture of digitalis and syrup of poppies were given in mixture daily. He remained in the infirmary about five months, and was made an out-patient on the 23d of February, 1829.

"After this time, I was in the habit of visiting him at his own house, for his sufferings were such, that he was unable to walk any distance without the greatest difficulty. The last time I had an opportunity of seeing him, the symptoms were all very materially increased. His nights were restless, with frequent orthopnœa; great soreness in the right side, aggravated on coughing; tumour more diffused, the pulsation being evident between the third and fourth ribs, but less vehement than at the intercostal space directly above; carotids much excited; constant head-ache; arm on same side painful, with a sense of numbness and loss of power; tickling cough; distressing rattle in the throat; expectoration neither bloody nor purulent; difficulty of lying down in bed, often obliged to get up and walk about his room from dyspnœa, and a sense of suffocation, occasioned by the recumbent posture; inclination to vomit, with frequent retching; bowels open; urine scanty and dark coloured; tongue with a slight brownish coat in the centre; pulse 112, rather full, regular; eyes glassy; great anxiety.

His chief relief was from the use of leeches, and these to the number of eight or ten, were very often applied."

On the 2d Aug. whilst making some inconsiderable exertion in the back yard, he fell down and expired in a few moments.

"*Sectio Cadaveris*. The body was by no means attenuated, but there was great pallor of the countenance and lips, indeed, throughout the whole cutaneous surface, there appeared a total want of blood in the capillaries. The chest, on percussion, emitted a good sound on the left side, but uniformly dull upon the right, especially around and below the situation of the tumour.

On opening the cavity of the thorax, an aneurism, of a very considerable size, was discovered involving the ascending portion of the arch of the aorta, implicating the arteria innominata, and extending as far as the origin of the subclavian on the left side, the former vessel seeming much dilated and thickened. The tumour extended anteriorly from the second to the fourth rib; but no absorption had taken place in the osseous or cartilaginous structure of either of these, or the intervening one, in contact with the disease. On cutting into the pericardium, a large quantity of serum was removed, and a coagulum of crassamentum was observed completely surrounding the heart, and forming, as it were, a mould of that organ. The pericardium, indeed, was literally filled and distended with the effused blood; in quantity, I should conceive at least twenty ounces. The heart, itself, *was strong and apparently healthy*; it may be, however, *somewhat larger than natural*. One of the aortic valves had a large process of ossific matter in it, the other two being perfectly free from any deposit of the kind. There was an opening, though partially closed by a plug of lymph, between the sac and the pericardium, through which the blood had escaped, of sufficient size to admit the passage of a small quill; it had no mark of ulceration, but gave to me the impression of a rent. The parietes of the sac were of unequal thickness, in some places an inch or more, in others, not exceeding the twelfth part of one. It was thickest at the front, where confined by the ribs, and this contained within its substance a second tumour, having much the character of a scrofulous gland in a state of suppuration, for on dividing it, a curdy or cheesy kind of matter escaped; it had no opening of communication with the sac. The trachea was much compressed by its contiguity to the disease, and was removed with it, the aneurism being attached by cellular membrane. Very extensive adhesions were likewise contracted both in the anterior and posterior parts of the thorax, and these, together with the flattened state of the trachea, must greatly have diminished the faculty of respiration.

A portion of the aorta below the tumour, being cut out and laid open, exhibited its coats in an unhealthy state; they were generally much *inflamed* and thickened, the internal one being thrown into rugæ, with here and there numerous irregular depositions of ossific matter and *lymph;* the latter, when removed, showing a surface nearly approaching to ulceration."

The above is a very characteristic case. The pulsating tumour between the intercostal spaces to the right of the sternum indicated the existence of aneurism at the root of the aorta, and the tickling cough and slight dysphagia, with the preternatural pulsation of the carotids and heart symptoms, pointed out with almost unerring certainty more general affection of the arch and probably hypertrophy of the left ventricle. Those who are in the habit of studying cardiac disease on a large scale with the stethoscope could, to use a coarse expression, swear to the disease with their eyes shut. It is said in the notes of the dissection that the heart was "strong and apparently healthy; it might be somewhat larger than natural." Had the reporter been conversant with the diseases of this organ, we have no doubt but he would have discriminated hypertrophy of the left ventricle, for in four cases out of five of the aneurisms or dilatations of the aorta which we have witnessed, such has been the pathological condition of the heart. The profession in general are but very imperfectly acquainted with the almost universal connexion between disease of the heart and diseases of the arteries. In the notes of the sectio cadaveris, it is also stated that the coats of the aorta *below* the tumour were *inflamed*, and encrusted with *lymph*. This is a mistake, for neither is the thickening of the coats of arteries the result of inflammation, nor are the *végétation-like* incrustations on their interior coagulable lymph. The changes and deposites are the results of hard labour or old age or both, the lymph-like concretions are the fibrine of the blood, deprived merely of its colouring matter. Of these facts there can be no doubt, and the reporter's statements are misconceptions.

We have reason to believe that aneurismal dilatations or pouches of the aorta at its root, or even within the embrace of the pericardium, are not uncommon. We lately saw a dissection of such a case, we have another patient so affected under our care at the present time, and M. Cruveilhier in his recently published plates of morbid anatomy delineates a representation of a third. Add to this the case of which we have just given an account, and two or three others of which we have read, and the list, without any research, will be respectable. When tolerably far advanced the diagnosis is not difficult, but when only in their first or incipient stage, the true nature of the cases is not so easily made out. In the two instances that have fallen under our own observation there was the bruit de scie or de soufflet in the tumour, and more or less evidence of hypertrophy of the ventricle. When the disease has made some progress the characteristic symptoms or appearances are:—a pulsating tumour more or less extensive and prominent in the intercostal spaces immediately to the right of the sternum, generally between the second and the fourth ribs; a *bruit* in the tumour, and more action than natural in the heart; preternatural pulsation in the arteries of the neck; perhaps some difference of pulse in the upper extremities and numbness of the right; a certain degree of cough, dyspnœa, palpitation, and other usual symptoms of cardiac affection; a middle or advanced age; and a history corroborating the suspicion of aneurism. We have known such an affection in its early stage considered as bronchitis, and we have seen an aneurismal dilatation of the aorta and innominata pressing on the trachea, overlooked and treated for the same; but we are sure that a physician in the habit of employing the stethoscope, and *understanding* it, will not make one such blunder, where another who does not employ it makes five.

With regard to the treatment we have little to say. Rest and the alternation of the mild narnotics, as conium, hyosciamus, poppy and lettuce, with a diet below par, and occasional *small* bleedings, appear to us to be productive of more benefit in the great majority of cases both of aneurism and organic disease of the heart, than heroic treatment whether it be bleeding or starvation. The first almost certainly drives on a dropsy, and we really believe that the latter has been carried by practitioners much too far. With regard to the exhibition of digitalis, we have only to say that we are afraid of it, for in the hands of those who have used it freely, we *know* that it has not been an unusual thing for their patients to have died most suddenly and unexpectedly. More ghosts than one have been guided to the "shores of Acheron," by the caduceus of foxglove. We know not what to say of counter-irritants, except that we have seldom seen them of service, and at times have thought that they did mischief. Leeches are occasionally useful, but they should not be placed upon the tumour, in fact the practitioner should always apply his counter-irritants, if he uses them, in the vicinity only of the disease, for nothing can be more pernicious than leeching and blistering the skin directly over an aneurism. We believe that more benefit is derived from plasters of belladonna or stramonium than from any applications of the former class. Of course the flying attacks of inflammation that occasionally come upon the pleura, the lung, or the pericardium, must be met at the time by appropriate, but not violent measures. Had we space to dilate on this topic, to us a very interesting one, we might hope to draw the attention of our brethren to some curious and some useful points. Here, however, we must stop, and our only excuse for having ventured on these, already tedious, observations is, that we have really seen a good deal of this class of affections, and we do not speak from random observation, theoretic notions, or bookish experience.

